# Comparison of environmental and isolate *Sulfobacillus* genomes reveals diverse carbon, sulfur, nitrogen, and hydrogen metabolisms

**DOI:** 10.1186/1471-2164-15-1107

**Published:** 2014-12-15

**Authors:** Nicholas B Justice, Anders Norman, Christopher T Brown, Andrea Singh, Brian C Thomas, Jillian F Banfield

**Affiliations:** Department of Earth and Planetary Science, University of California, Berkeley, CA 94720 USA; Physical Biosciences Division, Lawrence Berkeley National Lab, Berkeley, CA USA; Section for Infection Microbiology, Department of Systems Biology, Technical University of Denmark, Lyngby, Denmark

## Abstract

**Background:**

Bacteria of the genus *Sulfobacillus* are found worldwide as members of microbial communities that accelerate sulfide mineral dissolution in acid mine drainage environments (AMD), acid-rock drainage environments (ARD), as well as in industrial bioleaching operations. Despite their frequent identification in these environments, their role in biogeochemical cycling is poorly understood.

**Results:**

Here we report draft genomes of five species of the *Sulfobacillus* genus (AMDSBA1-5) reconstructed by cultivation-independent sequencing of biofilms sampled from the Richmond Mine (Iron Mountain, CA). Three of these species (AMDSBA2, AMDSBA3, and AMDSBA4) have no cultured representatives while AMDSBA1 is a strain of *S. benefaciens,* and AMDSBA5 a strain of *S. thermosulfidooxidans*. We analyzed the diversity of energy conservation and central carbon metabolisms for these genomes and previously published *Sulfobacillus* genomes. Pathways of sulfur oxidation vary considerably across the genus, including the number and type of subunits of putative heterodisulfide reductase complexes likely involved in sulfur oxidation. The number and type of nickel-iron hydrogenase proteins varied across the genus, as does the presence of different central carbon pathways. Only the AMDSBA3 genome encodes a dissimilatory nitrate reducatase and only the AMDSBA5 and *S. thermosulfidooxidans* genomes encode assimilatory nitrate reductases. Within the genus, AMDSBA4 is unusual in that its electron transport chain includes a cytochrome *bc* type complex, a unique cytochrome *c* oxidase, and two distinct succinate dehydrogenase complexes.

**Conclusions:**

Overall, the results significantly expand our understanding of carbon, sulfur, nitrogen, and hydrogen metabolism within the *Sulfobacillus* genus.

**Electronic supplementary material:**

The online version of this article (doi:10.1186/1471-2164-15-1107) contains supplementary material, which is available to authorized users.

## Background

Species of the *Sulfobacillus* genus are Gram-positive spore forming bacteria belonging to the order Clostridiales, and are found globally in acidic environments such as thermal springs [1], hydrothermal vents [2], solfatara fields [3], and acid mine drainage (AMD) environments [4]. *Sulfobacillus* species are also frequently found in industrial reactors used in bioleaching operations [5]. As such, an understanding of their physiology is of both environmental and biotechnological importance.

In the AMD environment of the Richmond Mine (Iron Mountain, CA) *Sulfobacillus* are members of acidophilic, metal-tolerant microbial consortia that promote sulfide-mineral dissolution. In contrast to the dominant iron-oxidizing *Leptospirillum* bacteria, or certain members of the heterotrophic *Thermoplasmatales* archaea, *Sulfobacillus* generally appear in much lower relative abundances, having been identified only through 16S rRNA clone libraries, fluorescent in situ hybridization [6–8], and as unresolved fragments assembled from metagenomic sequence information [9]. As their namesake implies, they are likely key players in sulfur cycling, yet relatively little is known about their metabolic pathways or how these vary across the *Sulfobacillus* genus.

Key features of *Sulfobacillus* metabolism have been described for several isolated *Sulfobacillus* species—*S. sibiricus* [10] *, S. thermotolerans* [11], *S. acidophilus* [1] *, S. thermosulfidooxidans* [12] *,* and *S. benefaciens* [13] *.* Each is a facultative anaerobe capable of assimilation of organic and inorganic forms of carbon, deriving energy from aerobic oxidation of iron and various sulfur species (e.g., tetrathionate and elemental sulfur), as well as from ferric iron respiration [10, 11, 14, 15], and possibly fermentation [15]. Other phenotypic differences such as metal tolerance, temperature tolerance, and use of different carbon compounds set these organisms apart [5, 11, 13]. Genomes for two of these species—*S. acidophilus* and *S. thermosulfidooxidans*—have been previously reported [2, 16–18] and an additional *S. thermosulfidooxidans* genome is deposited in the publicly available “Integrated Microbial Genomes and Metagenomes” (IMG) database [19]. Here we present five new draft genomes of *Sulfobacillus* organisms assembled from metagenomic data. We analyze these genomes in the context of the previously published genomes of *Sulfobacillus* isolates, and describe key elements of carbon, sulfur, nitrogen, and hydrogen metabolism. In addition to illuminating the metabolic potential of a strain of *S. benefaciens,* and three other novel *Sulfobacillus* spp., this work provides one of the first genome-inferred reconstructions of Gram-positive, acidophilic sulfur oxidation pathways, and highlights both conserved and divergent metabolisms found in organisms of the *Sulfobacillus* genus.

## Methods

DNA was collected from the 5way site at Iron Mountain Mine [20] and extracted as previously described [21]. This sample is referred to as ‘5way fungal streamer,’ and comprised the majority of sequencing information used in the genome assembly. Briefly, 4–5 ml of frozen biofilm was washed and thawed with cold acidified 0.9% NaCl (pH 1.0, H_2_SO_4_), homogenized with a 16 G needle in cold pH 7.0 phosphate buffered saline, and finally ground in a mortar and pestle in liquid nitrogen. The powdered biofilm pellets were then extracted with a PowerSoil DNA isolation kit (MoBio Laboratories, Carlsbad, CA, USA). Illumina library preparation sequencing was carried out according to JGI protocols [21, 22].

Three flow cells of Illumina HiSeq were used to obtain ~90 million paired-end 76 bp reads (8.46 Gb). An additional overlapping paired-end library was sequenced to generate longer sequence reads. The initial paired-end reads were merged using SeqPrep [23] into ~32 million long reads (6.17 Gb) with a median length of 194 bp. Reads from 17 additional previously published metagenomic datasets were used to assist binning and assembly [24].

### Metagenomic sequence assembly

A total of 854 million paired-end (PE) Illumina reads (98 Gb) pooled from the 18 samples described above were assembled *de novo* using the Iterative De-Bruijn Assembler optimized for single-cell and metagenomic assemblies (idba-ud) [25]. A kmer-range of 19–99 was used with the pre-correction option enabled. All PE-reads were quality-trimmed using Sickle (sickle pe options -q 15 and -l 40) prior to assembly. PE-reads were mapped back to the resulting scaffolds using bwa [26]. All scaffolds larger than 1500 bp were binned using emergent self-organizing maps (ESOM) trained on tetranucleotide frequencies as previously described [9]. Briefly, larger sequences were subdivided into 10 kb fragments, and trained for 100 epochs using the k-batch training method, along with any leftover fragments exceeding 3 kb in length. To minimize noise, sequences in the range 1500–3000 bp were not included during training, but instead projected onto the trained weight vectors generated with the larger fragments. In order to separate individual *Sulfobacillus* bins, a discrete bin containing putative members of the order Clostridiales, was isolated and further binned using log-normalized sample abundance patterns from 18 different samples, this time including fragments down to 1500 bp. Five distinct sub-bins (AMDSBA1, AMDSBA2, AMDSBA3, AMDSBA4 and AMDSBA5) were individually re-assembled *de novo* using every read-pair that mapped within these respective bins. Additionally, because of its high similarity to *S. thermosulfidooxidans* DSM9293, all PE-reads mapping to this organism were included in the AMDSBA5 sub-assembly as well. Due to its considerably lower abundance, AMDSBA2 was sub-assembled using PE-reads as described above, but with the inclusion of additional reads from the overlapping HiSeq PE library that mapped within AMDSBA2 bin (median read length 194 bp). Scaffolds belonging to the Clostridiales bin, but not to the 5 putative genome bins were not re-assembled. In order to identify areas of inconsistent coverage possibly indicative of chimeric assemblies, reads were mapped back to reassembled genomes and scaffolds binned a second time again using time-series abundance data. The resulting ESOM map was used to identify scaffold fragments that binned inconsistently, suggestive of chimeric assemblies. These scaffolds were manually checked with stringent paired read mapping (100% identity) to either confirm the assembly or identify points where chimeras might occur.

### Metabolic analysis

Open reading frames on assembled contigs for Iron Mountain *Sulfobacillus* and *S. thermosulfidooxidans* strain ‘Cutipay’ were predicted using Prodigal [27], and tRNAs predicted with tRNAscan [28]. Proteins were then compared with BLAST [29] to UniRef90 [30] and KEGG [31] with matches greater than 60 bits being reported. Reverse BLASTs were also used to identify reciprocal best BLAST hits. Proteins were further analyzed with InterProScan [32] to identify conserved domains. Protein sequences and corresponding annotations for *S. thermosulfidooxidans* AT-1 (DSM9293), *S. acidophilus* TPY, and *S. acidophilus* NAL (DSM10332) were downloaded from the Integrated Microbial Genomes database available at http://img.jgi.doe.gov/ [33]. While *S. thermosulfidooxidans* strain ‘Cutipay’ and *S. acidophilus* TPY were included in most analyses here, results focus on the high quality genomes of the type strains *S. thermosulfidooxidans* AT-I (DSM9293) and *S. acidophilus* NAL (DSM10332). Unless otherwise specified, all references to *S. thermosulfidooxidans* and *S. acidophilus* in the text refer specifically to these type strains.

Orthologs shared between species were identified with amino acid sequence searches between all pair-wise combinations of genomes using USEARCH [34]. Reciprocal best hits between each pair of genomes were considered orthologs if the alignments of their sequences had an E-value less than 0.01, a Bit score greater than 40, and covered at least 65% of each amino acid sequence.

### Protein phylogenetic analysis

All protein trees were made by first aligning protein sequences with MUSCLE [35]. Next, alignments were trimmed using Gblocks [36, 37], which stringently curates the alignment to phylogenetically informative sites. For each alignment, the optimum amino acid substitution model was estimated using ProtTest [38]. All trees were generated using RAxML [39] using the PROTCAT rate model and the ProtTest-determined amino acid substitution model. Support was evaluated using 500 bootstrap replications in RAxML. Trees were visualized in iTOL [40].

The concatenated ribosomal protein tree was made using 16 core ribosomal proteins (rpL2, 3, 4, 5, 6, 14, 15, 16, 18, 22, 24, rpS3, 8, 10, 17, 19), selected based on low frequencies of lateral gene transfer [41, 42]. These proteins were aligned with MUSCLE [35], manually trimmed, then concatenated, resulting in an 2,052 residue alignment. Phylogenetic relationships were analyzed using RAxML using the LG + *α* + *γ* substitution model, and nodal support determined with 500 bootstrap replicates. Trees were visualized in FigTree (available: http://tree.bio.ed.ac.uk/software/figtree/)

### 16S rRNA gene sequence analysis

Near full-length 16S rRNA sequences for *Sulfobacillus* species were generated using EMIRGE—an iterative template-guided assembler that probabilistically generates 16S rRNA gene sequences using a 16S rRNA database [21]. First, potential 16S rRNA gene regions were found by a BLAST of all assembled contigs against SILVA db v. 108 [43]. Reads that mapped to these regions were extracted and trimmed with Sickle (available https://github.com/najoshi/sickle), allowing only paired-end reads with length >60 and quality scores >20. For the reference database, 186 sequences were downloaded from the SILVA SSU database representing the 174 sequences of ‘Family XVII Incertae Sedis’ (the family to which the *Sulfobacillus* genus belong) as well as twelve representative sequences of other organisms known to be represent the majority of the Richmond Mine microbial communities, including *Leptospirillum* species and Archaea of the ARMAN and *Thermoplasmatales* lineages. Potential chimeric sequences in this database were removed with DECIPHER [44] and UCHIME [45] [searched against the 2011 Greengenes database [46]]. EMIRGE was run in 50 iterations with a “join_threshold” parameter of 0.99.

Using the QIIME software suite [47], the EMIRGE-generated sequences and representative sequences of *Sulfobacillus* isolates were aligned using PyNAST with default parameters [48], and filtered with *filter_alignment.py* in QIIME. Edges were trimmed leaving 1,154 unambiguously aligned positions. A maximum likelihood tree was generated with RAxML using the GTRCAT model. Nodal support was evaluated with 500 bootstrap replicates. Trees were visualized in FigTree.

### Microscopy

Environmental samples were fixed and stained with lineage-specific fluorescent *in situ* hybridization (FISH) probes to examine *Sulfobacillus* populations, as described previously [49]. *Sulfobacillus* (SUL230; 5’- TAATGGGCCGCGRGCYCC) and archaeal-specific probes (ARC915; 5’-GTGCTCCCCCGCCAATTCCT) have been previously reported [7, 50].

## Results

### Overall genome statistics and phylogeny

**Figure 1:**
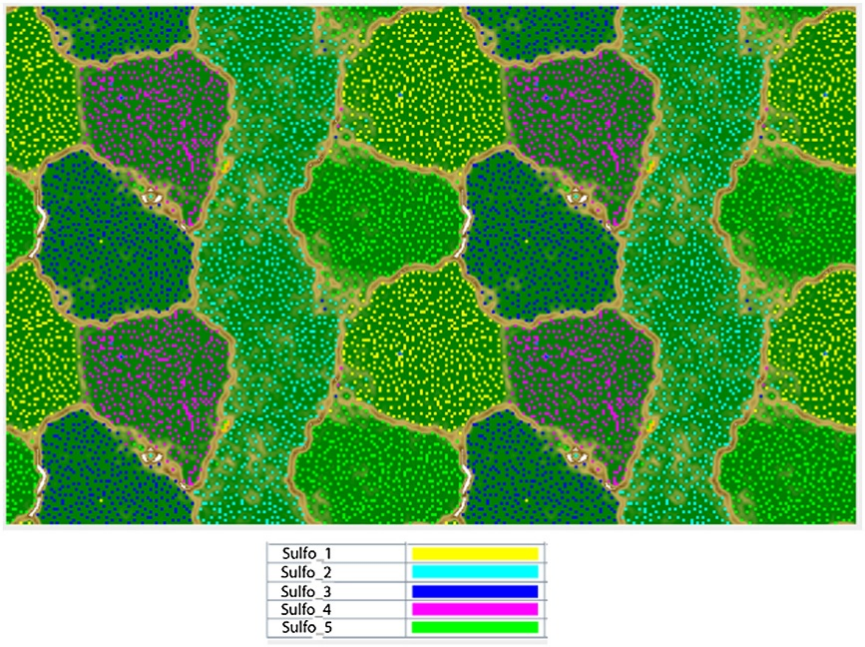
**Emergent self-organizing map (ESOM) of Clostridiales sequence fragments binned by time-series abundance patterns (>1500 bp).** Note that the map is continuous (top and bottom edges, left and right edges). Each point represents a sequence fragment with colors indicative of the genome bin it belongs too (see legend below).

We reconstructed the near complete genomes of five *Sulfobacillus* species from the Richmond Mine, represented as five discrete bins on abundance-pattern ESOMs (Figure 1). Each genome had between 129 and 409 scaffolds with coverage ranging from 10 to 258X (Table 1). Genomes were between 3.07 and 4.56 Mbp, and encoded between 3,312 and 4,629 protein-coding genes. Additional file 1: Figure S1 summarizes the completeness of each genome estimated using the presence of a set of conserved, single copy genes with low frequency of horizontal gene transfer [41, 42].

**Table 1:** **General statistics of the** ***Sulfobacillus*** **genomes**

Name	Genome size (Mbp)	GC Content (%)	Coverage (X)	Scaffolds	N50	Protein coding genes	Reference
AMDSBA1	4.56	52.19	258	155	65,127	4629	This study
AMDSBA2	3.07	52.37	10	409	10,877	3312	This study
AMDSBA3	3.68	55.54	113	129	49,240	3785	This study
AMDSBA4	4.11	51.67	25	179	63,277	4254	This study
AMDSBA5	3.65	49.15	92	161	65,096	3870	This study
*S. thermosulfidooxidans* AT-1 (DSM 9293)	3.86	49.64	N/D	2	-	3875	IMG JGI
*S. thermosulfidooxidans ‘*Cutipay’	3.86	49.30	116	35	509,367	3600	Travisany [16]
*S. acidophilus* NAL^T^ (DSM 10332)	3.56	56.75	168	1	-	3626	Anderson [17]
*S. acidophilus* ‘TPY’	3.55	56.70	26	1	-	3770	Li [2]

**Figure 2:**
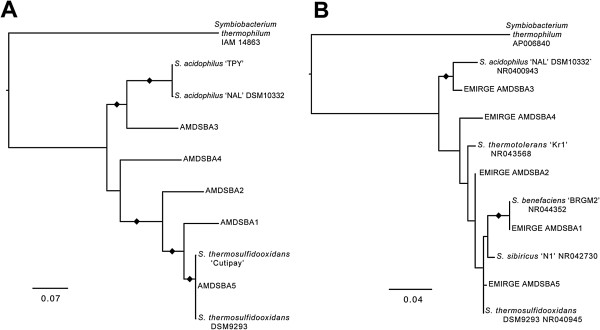
**Maximum likelihood phylogenetic trees showing relationship amongst Iron Mountain and published** ***Sulfobacillus*** **species using A) concatenated alignment of sixteen ribosomal proteins and B) EMIRGE-generated 16S rRNA genes.** Black diamonds indicate nodes with greater than 90% bootstrap support.

Based on phylogenetic trees of concatenated protein alignments and EMIRGE-generated 16S rRNA genes, AMDSBA5 was classified as a strain of *S. thermosulfidooxidans* (99.7% 16S rRNA gene similarity), AMDSBA1 was classified as a strain of *S. benefaciens* (100% 16S rRNA gene similarity), and AMDSBA3 was shown to related to be most closely related to *S. acidophilus* strains (97.2% 16S rRNA gene similarity, Figure 2A and B, Additional file 2: Table S1). AMDSBA2 may represent another strain of *S. thermosulfidooxidans* with 99.2% 16S similarity, while AMDSBA4 is at least 97% divergent from any *Sulfobacillus* isolates.

### *Sulfobacillus*abundance and distribution

Previous data have indicated the presence of *Sulfobacillus* species in low abundance in late growth stage and submerged biofilms [7], as well as in warmer (~43°C) areas of the Richmond Mine [6]. They are sometimes found to form compact structures of rope-like assemblages (Additional file 3: Figure S2), and similar filamentous-like growth as been reported elsewhere [1, 15]. Metagenomic sequencing data indicated that species AMDSBA1 and AMDSBA4 are most abundant in late-growth stage biofilms (Additional file 4: Figure S3). The higher abundances in the ‘5way fungal streamer’ sample may reflect the unique DNA-extraction procedure (grinding in liquid nitrogen) used on this sample alone, potentially improving lysis of Gram-positives and their vegetative spores.

### Energy metabolism: electron transport chain

**Figure 3:**
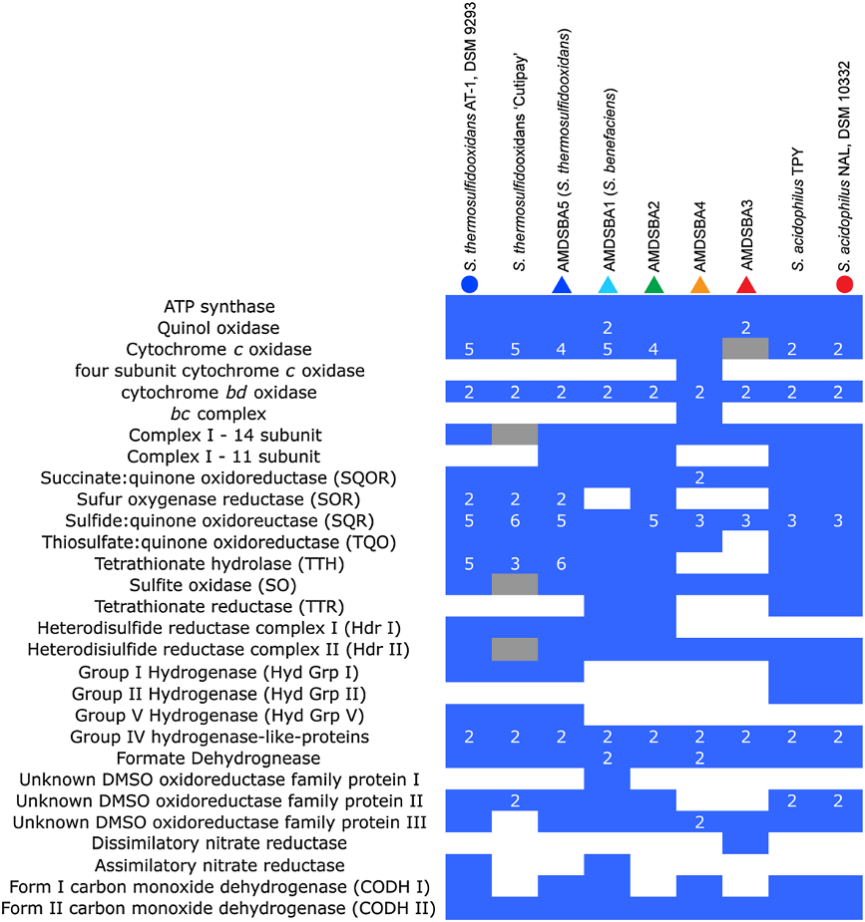
**Gene copy numbers for electron transport chain, sulfur, hydrogen, nitrogen metabolisms in** ***Sulfobacillus*** **genomes.** A filled blue square indicates presence, with a white number indicating copy numbers >1. Filled grey squares are presumed present due to presence in closely related organisms or partial gene identification. Colored circles and triangles at the top of the graph indicate species identifier in Figure 5.

Prior studies have shown that *Sulfobacillus* are capable of aerobic growth. Genomes indicate that oxygen reduction can occur via several different terminal oxidases. Each of the *Sulfobacillus* genomes analyzed in this study has genes encoding the four-subunit proton pumping quinol oxidases, although only a fragment of subunit II of the quinol oxidases from AMDSBA4 and AMDSBA2 were obtained. AMDSBA1 and AMDSBA3 contained an additional quinol oxidase (Figure 3, Additional file 5: Table S2), and this second copy may have a different affinity for oxygen. Except for AMDSBA3, all of the *Sulfobacillus* genomes also contain cytochrome *c* oxidase encoding-genes. The number of cytochrome *c* oxidase-encoding genes varies between species with *S. acidophilus* containing 2 copies, with *S. thermosulfidooxidans* strains containing at least 4 (Figure 3). Furthermore, a unique terminal oxidase from AMDSBA4 (AMDSBA4_44_9-12) was orthologous to the quinol oxidases of the other *Sulfobacillus* (and contained four subunits)*,* however it contained a Cu_A_-binding site in subunit II, indicating it accepts electrons from cytochrome *c* and not quinones, [51, 52]. Additionally, cytochrome *c*-binding sites (CXXCH) were identified in the C-terminal end of both subunits II and IV which are not present in any other terminal oxidases here, suggesting the electron transport chain of AMDSBA4 is distinct from other *Sulfobacillus* species.

AMDSBA4 is also unique in that it contains a cytochrome *bc* complex. Other *Sulfobacillus* have a di-heme cytochrome *b* homologous to the *petB* gene found in other cytochrome *bc* complexes, however the absence of cytochrome *c* and the iron sulfur protein in these operons suggests that these are not canonical *bc* complexes and may perform some other function.

*c*-type cytochromes are annotated in all genomes except AMDSBA1, although the presence of cytochrome *c* oxidases and cytochrome *c* assembly factors would indicate that *c*-type cytochromes are likely present.

All of the *Sulfobacillus* genomes contain multiple genes encoding the two-subunit *bd*-type quinol oxidases. The cytochrome *bd* respiratory oxygen reductases contribute to the proton motive force by transmembrane charge separation without a proton pump [53].

Each genome contains genes encoding a classic 14-subunit NADH:quinone oxidoreductase (Complex 1). In addition, an 11-subunit Complex 1 is conserved in AMDSBA1, AMDSBA2, AMDSBA5, and *S. acidophilus*. This 11 subunit gene cluster lacks genes encoding the “N-module” subunits (*nuoE*, *nuoF*, and *nuoG*) that are involved in NADH binding, and has been suggested to be the evolutionary ancestor of the 14 subunit complex [54, 55]. The lack of the N-module suggests it can receive electrons from donors other than NADH [54].

Genes encoding succinate:quinone oxidoreductases (SQORs) are found in all genomes. These SQORs are characterized by having a single hydrophobic subunit anchor with two heme groups, and thus can be classified as ‘Type B’ [56]. AMDSBA4, however, has an additional SQOR adjacent to the first (AMDSBA4_13_54-57), distinct in that it contains two hydrophobic subunit anchors, each predicted to bind a heme group. As such, it is most similar to ‘Type A’ SQOR enzymes commonly associated with Archaea [56, 57]. The functional significance of this second SQOR in AMDSBA4 is unclear.

### Sulfur metabolism

Oxidation of sulfur, tetrathionate, and sulfide minerals is a well-documented characteristic of *Sulfobacillus* spp. [5, 10, 11, 13], and the genomes analyzed here are replete with enzymes involved in sulfur oxidation. In the AMD environment, elemental sulfur and various polythionates such as tetrathionate and pentathionate are ultimately formed by the reaction of acid-insoluble sulfide minerals (e.g., pyrite) with ferric iron [58–60].

Aerobically, *Sulfobacillus* spp. may oxidize sulfur in a disproportionation reaction using sulfur oxygenase reductase (SOR), which disproportionates linear polysulfide in the presence of oxygen to sulfite and hydrogen sulfide [61]. SOR is found in *S. acidophilus*, AMDSBA2, and in two copies in both S*. thermosulfidooxidans* and AMDSBA5 (Figure 3, Additional file 5: Table S2). Best studied in the archaeon *Acidianus ambivalens* [61], the SOR protein is cytoplasmic, and the lack of cofactors means it is not coupled to electron transport, supported by work done in *Acidithiobacillus caldus* [62]. All *Sulfobacillus* SOR proteins possess the identified conserved residues for a functional enzyme [61, 63].

Another possible route of sulfur compound oxidation may be through heterodisulfide reductase-like (Hdr) proteins encoded in all *Sulfobacillus* genomes. In methanogens, heterodisulfide reductases reduce heterodisulfide bonds in the final step of methanogenesis [64]. However, proteins related to heterodisulfide reductases are widely distributed and are suspected to be involved more generally in various forms of sulfur metabolism [65, 66]. Clusters of Hdr-like proteins are conserved in other acidophilic sulfur oxidizing Bacteria and Archaea and are upregulated during aerobic growth on sulfur in *Aciditothiobacillus ferroxidans*, where they are predicted to oxidize disulfide intermediates to sulfite [67]. The proposed disulfide substrates are sulfane sulfur species (RSS_n_H), formed by the reaction of cellular thiols (e.g., cysteine residues) with extracellular elemental sulfur [68, 69].

**Figure 4:**
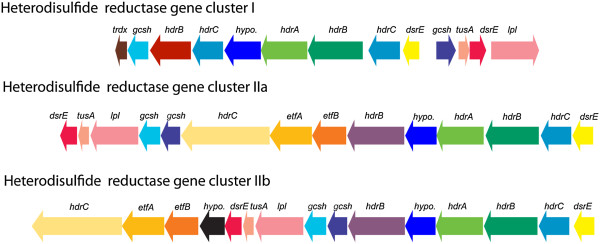
**Operon structure of putative heterodisulfide reductase complexes.** Hdr cluster I is found in *S. thermosulfidooxidans*, AMDSBA1, AMDSBA2, and AMDSBA5. Hdr cluster IIa is found in *S. thermosulfidooxidans* AMDSBA1, AMDSBA2, AMDSBA4, and AMDSBA5. Hdr cluster IIb is found in *S. acidophilus* and AMDSBA3. Gene abbreviations are as follows: thdx, thioredoxin; gcsh, glycine cleavage system protein H; hdrABC, heterodisulfide reductases; hypo, hypothetical; dsrE, dsrE-like sulfur relay protein; tusA, sulfur transfer protein; lpl, lipoate protein ligase; etfAB, electron transfer flavoprotein. Gene colors correspond to protein orthology as defined in methods.

Two different types of *Sulfobacillus hdr* gene clusters were found with varying gene content (Figure 4). One group, *hdr* gene cluster I, was identified only in *S. thermosulfidooxidans,* AMDSBA1 and AMDSBA5. This cluster includes an HdrA-like protein with an FAD-binding site and a conserved-cysteine motif (CxGCRDx_6-8_CSx_2_CC) typical in binding an Fe-S cluster. Two HdrB proteins are present which contain the cysteine-rich CCG domains (Cx_n_CCGx_m_C) predicted to bind [4Fe-4S] clusters [70]. The HdrC protein contains another [4Fe-4S] binding center. Furthermore, two DsrE-family proteins and a TusA homolog are encoded in this locus. DsrE and TusA proteins are strongly implicated in persulfidic sulfur (RS-SH) transfer in *Allochromatium. vinosum* [71, 72] *.* It is also interesting to note that a homolog to the glycine cleavage protein H (a lipoic acid containing protein) is found in this locus as is a lipoate-protein ligase that catalyzes the attachment of lipoic acid to target proteins [73]. The lipoic acid moiety of glycine cleavage protein H acts as a “swinging arm” that serves to transfer reaction intermediates between the various catalytic sites of the other proteins in the glycine cleavage system complex [74]. It is tempting to speculate that lipoic acid, with its disulfide bond, may play a key role in sulfur species transfers amongst this heterodisulfide complex, as has been noted by others [75].

The second group (*hdr* gene cluster II) is found in all *Sulfobacillus* genomes and contains many of the same subunits as *hdr* gene cluster I (Figure 4). However, there are additional genes encoding electron transfer flavoproteins as well as a second, longer *hdrC* gene containing 6 transmembrane helices and 2 cysteine-rich CCG domains not found in the HdrC of *hdr* gene cluster I. These three genes are syntenous, and similar arrangements have been observed in the sulfate reducing bacterium *Desulfobacterium autotrophicum* [76].

After SOR catalyzes the disproportionation reaction of linear polysulfide to H_2_S and sulfite, the sulfide may be oxidized back to sulfur by the membrane-bound sulfide:quinone oxidoreductases (SQR), with the concomitant reduction of quinones. Thus, sulfur disproportionation might be linked to the electron transport chain [77, 78]. Across the *Sulfobacillus* genomes, 74 proteins were annotated as SQRs or more generally as “FAD-dependent pyridine nucleotide disulfide oxidoreductases,” the family to which SQR proteins belong. There are three active-site cysteine residues shown to be important for SQR activity, with the second and third being essential [77, 79]. Alignment of these 74 *Sulfobacillus* putative SQRs and their related oxidoreductases with the characterized SQR of *Acidothiobacillus ferroxidans* showed 9 as having all three active-site cysteine residues, and 27 as having the second and third essential active-site cysteine residues. Protein trees constructed with those 36 *Sulfobacillus* proteins containing at least the second and third essential active-site cysteine residues indicated that all proteins belonged to either Group III or Group V SQRs, based on the protein phylogeny laid out by Marcia et al. [77] (Additional file 6: Figure S4). Both groups have representatives with demonstrated sulfide:oxidase activities [77, 80]. Based on this analysis, SQR is likely present in the published genomes of all *Sulfobacillus* species analyzed here.

Tetrathionate can be disproportionated by tetrathionate hydrolase (TTH) into sulfate, thiosulfate, and other sulfur compounds [81–83]. TTH activity has been detected in a strain of *S. thermosulfidooxidans* [84], and tetrathionate oxidation is widespread amongst *Sulfobacillus* isolates [85]. TTH homologs were found in all but AMDSBA3 of the *Sulfobacillus* genomes (Figure 3, Additional file 5: Table S2). Based on TTH-family phylogeny laid out by Protze et al. [86], only *S. thermosulfidooxidans, S. acidophilus,* and AMDSBA1 have proteins that fall within characterized TTH-family clades (Additional file 7: Figure S5). The remaining enzymes belong to yet uncharacterized families of TTH homologs. Furthermore, TTH proteins have been shown to be periplasmic in Gram-negative organisms [[83] and refs therein], and thought to be extracellular in the archeaon *Acidianus ambivalens* [86], and thus may also be extracellular in *Sulfobacillus* species.

Membrane-associated thiosulfate:quinone oxidoreductase (TQO) enzymes were found in all *Sulfobacillus* genomes analyzed except for AMDSBA3 (Figure 3, Additional file 5: Table S2). TQO may oxidize thiosulfate formed by TTH back to tetrathionate and concomitantly passing electrons into the electron transport chain via the quinone pool [87].

Another mechanism of tetrathionate metabolism could occur via putative tetrathionate reductases (TTR), an enzyme belonging to the dimethylsulfoxide oxidoreductase (DMSOR) family of proteins with a molybdo-bis-pyranopterin guanine-dinucleotide cofactor [88]. Several *Sulfobacillus* DMSOR catalytic subunits clustered with a branch containing tetrathionate reductases, including the characterized tetrathionate reductase from *Salmonella enterica* (Additional file 8: Figure S6). In *S. enterica*, TTR is a three-subunit enzyme that can reduce trithionate and tetrathionate to thiosulfate and sulfite [89]. TTR proteins were found in *S. acidophilus,* AMDSBA1, and a partial sequence was available from AMDSBA2. Except for a second putative TTR in AMDSBA1 (AMDSBA1_3_25, which also lacks the second and third subunits), the putative TTR first and second subunits each contain a Tat-signal sequence, indicating an extracellular location. No obvious heme or quinone-binding subunits were observed in the membrane-anchor subunit, leaving questions as to how, or if, this enzyme interacts with an electron transport chain of the cell. Furthermore, we note that no tetrathionate reducing activity has been observed for any *Sulfobacillus* species, and it is impossible to say what the function of this enzyme is based on genomic inference alone at this time.

Other catalytic subunits of *Sulfobacillus* DMSOR-family proteins clustered in a branch containing the sulfur reductase of *Aquifex aeolicus* (SreA) as well as the sulfite-oxidizing protein of *Allochromatium vinosum* (SoeA, Additional file 8: Figure S6). The three-subunit sulfur reductase complex in *A. aeolicus* is suggested to be involved in the reduction of tetrathionate, sulfur, and polysulfide coupled to H_2_ oxidation via a quinone pool [90]. On the other hand, the three-subunit sulfite-oxidizing complex in *A. vinosum* couples quinone reduction to sulfite oxidation [91]. Similar three-subunit gene operons are found in all *Sulfobacillus* species analyzed here, and as no sulfur-compound reduction has yet been observed for any *Sulfobacillus* species, we believe that the *Sulfobacillus* proteins are likely sulfite-oxidizing proteins akin to the SoeABC proteins of *A. vinosum*. The *Sulfobacillus* proteins lack signal peptides, and thus, like the SoeABC complex in *A. vinosum*, may be cytoplasmically located and anchored to the membrane*.*

Sulfate assimilation may occur in *S. thermosulfidooxidans*, *S. acidophilus*, AMDSBA1, and AMDSBA3 by the combined action of an ATP sulfurylase, an adenosine 5’-phosphosulfate (APS) reductase, a ferredoxin-dependent sulfite reductase, and an O-acetylserine sulfhydrolase. The APS-reductase is homologous to 3’phosphoadenosine 5’-phosphosulfate (PAPS) reductases often found in sulfate assimilation pathways, but the presence of an iron-sulfur binding subunit suggests the preferred substrate is APS [92, 93]. AMDSBA5 lacked the ATP sulfurylase and APS reductase, thus inorganic sulfur assimilation may proceed from sulfite. Furthermore, AMDSBA2 and AMDSBA4 did not contain the sulfite reductase, which may reflect incomplete genomic coverage or point to inorganic sulfur assimilation directly from H_2_S.

### Iron oxidation and reduction

Many *Sulfobacillus* isolates are demonstrated iron oxidizers [1, 10, 11, 13]. In other acidophilic microorganisms, outer-membrane cytochromes have been implicated in the oxidation of iron, including in the Gram-negative bacteria *Leptospirillum* spp. and *Acidothiobacillus ferroxidans* [[94] and refs therein]. Electrons from iron are ultimately transferred to either a terminal oxidase complex to reduce oxygen and consume and pump protons (“downhill”) or to an NADH dehydrogenase (“uphill”) [94]. There are membrane-associated *c*-type cytochromes that could be involved in iron oxidation in all *Sulfobacillus* species examined here except AMDSBA1. Electrons could be transferred from these membrane *c*-type cytochromes to the terminal cytochrome *c* oxidases in a “downhill” oxidation of iron. The “uphill” reactions require harnessing the proton-motive force in order to push the electrons in a thermodynamically unfavorable direction, and this is accomplished by the *bc* complex in *Leptospirillum* spp. and *A. ferroxidans* [94]. Only AMDSBA4 is predicted to have this complex, and it would be interesting to know if it functions in reverse electron transport as in the Gram-negative iron oxidizers. Furthermore, genes encoding putative sulfocynanin proteins have been identified in a strain of *S. thermosulfidooxidans* (*S. thermosulfidooxidans* ST) and shown to be highly expressed during growth on ferrous sulfate [18]. The extent to which these may be involved in iron oxidation is unclear.

All isolate species of *Sulfobacillus* have been shown to reduce ferric iron [10, 11, 13, 14]. Although Fe^3+^ reduction is widespread in acidophilic microorganisms [95, 96], little is known about the precise enzymes and pathways involved. In *A. ferroxidans,* a *c*-type cytochrome has been implicated in ferric iron respiration [97] as has a rusticyanin [98]. Rusticyanin proteins are found annotated only in *S. thermosulfidooxidans* and AMDSB5, and membrane-bound *c-*type cytochrome proteins are found in all *Sulfobacillus* genomes except AMDSBA1. Osorio et al. [99] also proposed a model for ferric iron reduction in *A. ferroxidans* when elemental sulfur was an electron donor, including one in which Fe^3+^ was indirectly reduced by H_2_S generated from anaerobic sulfur disproportionation. This H_2_S ‘scavenging’ allows normally endergonic sulfur disproportionation to become thermodynamically favorable [100, 101]. While generation of hydrogen sulfide may play a role in ferric iron reduction, *Sulfobacillus* have shown to reduce iron in cultures without any sulfur species present [13, 102], implying that some mechanism for the direct reduction of iron is also present.

### Hydrogen metabolism

The catalytic subunits for twenty-seven different nickel-iron (NiFe) hydrogenase proteins were identified across all *Sulfobacillus* species, which cluster into 5 distinct subfamilies (Additional file 9: Figure S7). One conserved 16-subunit gene cluster was identified within *S. acidophilus, S. thermosulfidooxidans*, and AMDSBA5, and the catalytic subunit was found to cluster with Group 1 respiratory-uptake [NiFe]-hydrogenases (Additional file 9: Figure S7). Like other Group 1 hydrogenases, the *Sulfobacillus* hydrogenases possess a Tat-motif at the N-terminus of the small subunit and are thus predicted to be located extracellularly. There, they may oxidize H_2_, transferring protons to the quinone pool via a cytochrome *b*-containing subunit [103]. The second 16-subunit hydrogenase gene cluster was identified in *S. acidophilus* and *S. thermosulfidooxidans* species, although the operon structure and catalytic subunits differ (Additional file 5: Table S2). In *S. acidophilus*, the catalytic subunits share the greatest homology with subgroup A of the Group 2 hydrogenases (Additional file 9: Figure S7). Group 2 hydrogenases are cytoplasmic hydrogen-utilizing complexes most commonly associated with nitrogen fixing cyanobacteria [103]. The hydrogenases of *S. thermosulfidooxidans*, however, are most similar to a relatively recently identified fifth group of hydrogenases [Additional file 9: Figure S7, [104]]. This fifth group is composed of putative high-affinity hydrogenases and has been detected predominantly in Actinobacteria, Acidobacteria, and Chloroflexi [105]. Neither the hydrogenases of Group 2 nor Group 5 contain a signal peptide, and are thus predicted to remain in the cytoplasm. Moreover, they do not contain the *b*-type cytochrome subunit thought to aid in electron transfer to quinones, and the electron acceptor is unclear. No Group 1, 2 or 5 hydrogenases were found in AMDSBA1-4.

The catalytic subunits of the remaining hydrogenases indicate that they belong to the Group 4 hydrogenase family, and at least one representative is found in each *Sulfobacillus* species (Additional file 9: Figure S7). Six syntenic genes are found near each catalytic subunit (Additional file 5: Table S2), and bear homology to Complex I enzymes (nuoH, nuoL) and formate hydrogen-lyase enzymes (HyfE and HyfB). The catalytic large subunits of these hydrogenases lack the binding site motifs normally found in [NiFe] hydrogenases [N-terminal: RxCGxCxxxH; C-terminal DPCxxCxxH/R; [106]]. As such, these enzymes are similar to energy-converting hydrogenase-related complexes (Ehr) and their function remains unknown [103, 107, 108].

### Carbon monoxide dehydrogenases

Aerobic-type carbon monoxide dehydrogenases (CODH) of both Form 1 and Form 2 were identified in *Sulfobacillus* genomes (Figure 3, Additional file 5: Table S2). Form 1 CODHs are known to be involved in carbon monoxide oxidation to CO_2_ with release of reducing equivalents introduced into the electron transport chain usually via cytochromes [109]. Form I enzymes were found in all organisms except AMDSBA2 and AMDSBA3. The function of Form II enzymes—differentiated by the active site motif AYRGAGR in place of the Form I motif AYXCSFR—are not fully understood, and may also play roles in CO oxidation as well as oxidation of other substrates [109]. Form II CODH enzymes are found in all *Sulfobacillus* species.

### Nitrogen metabolism

AMDSBA3 is the only *Sulfobacillus* species analyzed here that contains genes for dissimilatory nitrate reduction, observed as a single *NarGHJI* operon (Additional file 8: Figure S6, Figure 3). No other components for denitrification or nitrite reduction were found. AMDSBA3 is thus the only *Sulfobacillus* species analyzed here predicted to be capable of using nitrate as an alternative electron acceptor in anaerobic environments.

AMDSBA1 and *S. thermosulfidooxidans* contained genes encoding assimilatory nitrate reductases (Figure 3, Additional file 5: Table S2). The proteins encoded by the nitrate reductase genes (*NasC*) are members of the DMSOR family of enzymes, and phylogenetically cluster near the assimilatory nitrate reductase of *Bacillus subtilis* (Additional file 8: Figure S6). However, unlike the nitrate reductase of *B. subtilis,* the nitrate reductases in AMDSBA1 and *S. thermosulfidooxidans* do not possess an electron transfer subunit (*NasB*) required for the transfer of electrons from NADH to nitrate [110], making the electron donor unclear (e.g., NADH, flavodoxin, or ferredoxin). Nearby genes encoding nitrite reductases (*NasDE*) in both species contain the necessary siroheme and NADH binding sites required for the six-electron reduction of nitrite to ammonia using NADH as the electron donor [110, 111]. Copper-containing nitric oxide-forming nitrite reductases were found in *S. acidophilus* strains, as were nitric oxide dioxygenase enzymes, which may oxidize nitric oxide to nitrate as a detoxification mechanism [112]. It has also been suggested that these enzymes may reduce nitric oxide to nitrous oxide anaerobically, which if true, would allow denitrification activity (nitrite to nitrous oxide) in these organisms [113].

Ammonium transporters were identified in all genomes, and ammonia can be assimilated into central metabolic pathways via glutamate dehydrogenase, glutamine synthetase and glutamate synthase.

### Autotrophy

*Sulfobacillus* spp. are known autotrophs, and published genomes of *S. thermosulfidooxidans* DSM 9293*,* and *S. acidophilus* indicate the presence of carbon fixation via Calvin-Benson-Bassham (CBB) cycle [2, 16, 17, 114]. All *Sulfobacillus* also have complete CBB cycle genes, including a Type I ribulose-1,5-bisphosphate carboxylase-oxygenase (RuBisCO). In addition to the Type I RuBisCO, AMDSBA4 and *S. acidophilus* strains have additional Type 4 RuBisCO-like proteins (Additional file 10: Figure S8) that likely participate in the methionine salvage pathway [115]. Interestingly, AMDSBA4 is the only genome with an annotated carboxysome (AMDSBA4_48_6-9).

### Central carbon pathways

In addition to autotrophic growth, *Sulfobacillus* isolates have been reported to grow mixotrophically and heterotrophically on a variety of carbon substrates, including glucose, fructose, glycerol and other various organic carbon compounds [11, 14, 116]. All organisms except *S. thermosulfidooxidans*, AMDSBA5, and AMDSBA1 have a complete Embden-Meyerhoff pathway for glycolysis. *S. thermosulfidooxidans*, AMDBA5, and AMDSAB1, lack a 6-phosphofructokinase. While genes encoding 6-phosphofructokinase could not be identified in *S. thermosulfidooxidans,* cell-free enzymatic assays suggest these cells can carry out this transformation [102]. Glucose-6-phosphate isomerase activity in AMDSBA4 and both *S. acidophilus* can be accounted for by the inclusion of unique bifunctional transaldolases/phosphoglucose isomerase [117]. The oxidative portion of the pentose phosphate pathway (glucose dehydrogenase, 6-phosphogluconolactonase, and 6-phosphogluconate dehydrogenase) was present in all organisms.

Complete semi-phosphorylative Entner-Doudoroff carbon degradation pathway (2-keto-3-deoxygluconate 6-phosphate aldolase, 2-dehydro-3-deoxygluconokinase, and gluconate dehydratase) was present in *S. acidophilus* strains, AMDSAB1, AMDSBA3, and AMDSBA4 (Additional file 5: Table S2). While no gluconate dehydratases were annotated, deoxy-acid dehydratases likely carry out this function as they do in other bacteria [118]. Phosphogluconate dehydratases, indicative of the phosphorylative Entner-Doudoroff pathway, were not found in any genomes. Enzyme assays from cell-free extracts of *S. thermosulfidooxidans* and *S. sibiricus* strains have shown this phosphogluconate dehydratase activity, however, as well as keto-3-deoxy-6-phosphogluconate aldolase activity *S. thermosulfidooxidans*, which would indicate a phosphorylative Entner-Douroroff pathway [102, 119].

Pyruvate can be oxidatively decarboxylated to acetyl-CoA with pyruvate dehydrogenase (found in all *Sulfobacillus*) or in AMDSBA1 with a putative pyruvate ferredoxin oxidoreductase (AMDSBA1_57_11-13). Excepting a few gaps from the low-coverage genome of AMDSBA2, the tricarboxylic acid cycle was complete in all genomes. Furthermore, a putative oxoglutarate synthase that may allow oxidations of 2-oxoglutarate to be coupled to the reduction of ferredoxin was found in all *Sulfobacillus* genomes. Finally, a complete glyoxylate bypass system (malate synthase and isocitrate lyase) was found in only AMDSBA1 and *S. acidophilus*, possibly permitting carbon assimilation of C_2_ compounds like acetate. Malate synthase was found in all but AMDSBA3 and AMDSAB4, which together with glycolate oxidase, may provide a route of glycolic acid assimilation. Glycolic acid is produced as an exudate by key primary producing acidophiles like *Leptospirillum*, and the degradation of glycolic acid sets *Sulfobacillus* apart amongst other acidophiles. In fact, it is thought to contribute to an organic-carbon degrading niche in stirred-tank bioleaching systems [120]. Lactate utilization proteins similar to those found in *Bacillus subtilis* were identified in all organisms, and are thought to allow growth on lactate [121].

### Formate dehydrogenase

Putative cytoplasmic selenocysteine-containing formate dehydrogenases are found in all *Sulfobacillus* genomes (Additional file 8: Figure S6). These genes possess the binding site and three catalytic residues necessary for formate oxidation. Each catalytic subunit is syntenous to cluster of genes with homology to the NADH-binding NuoEFG subunits, and thus may represent coupling of formate oxidation to NADH reduction.

### Fermentative metabolism

Strains of *S. thermosulfidooxidans*, *S. sibiricus*, and *S. thermotolerans* have been shown to produce propionate and acetate in the growth media during growth in hypoxic conditions [15]. Complete fermentative pathways that result in propionate formation, however, could not be identified. In all *Sulfobacillus* species, neither the enzymes lactoyl-CoA dehydratase [of the acrylate pathway [122]] nor propionyl-CoA:succinate-CoA transferase and methylmalonyl-CoA carboxyltransferase [of the succinate pathway for propionate fermentative pathway [123]] could be identified. Acetate production could be produced with substrate level phosphorylation by a phosphate acetyltransferase (found only in AMDSBA2) and an acetate kinase (identified in AMDSBA1 and AMDSBA2). Traditional fermentative pathways thus cannot account for propionate and acetate produced in isolate strains.

### Lipid and lipopolysaccharide biogenesis

All *Sulfobacillus* species have the genes required to synthesize fatty acids. Species of *Sulfobacillus* have been shown to contain predominantly branched-chain anteiso and ω-alicyclic fatty acids [124]. In general, branched chain fatty acids can be synthesized from either branched short-chain carboxylic-acid CoA esters or from α-keto acids [125]. In the *Sulfobacillus,* the former pathway is probably used, as branched chain α-keto acid decarboxylases were not identified. The chorismate pathway and genes encoding biosyntheis of ω-alicyclic fatty acids were identified in all *Sulfobacillus* species. The chorismate pathway produces shikimic acid as an intermediate [126], which is ultimately used to form cyclic carboxylic acid-coA esters used in the synthesis of ω-alicyclic fatty acids. Peptidoglycan biosynthesis was accounted for in all *Sulfobacillus*. An S-layer has been reported to be present on some cells of *S. thermosulfidooxidans* [127], however only a single annotated S-layer protein was found in AMDSBA1 (AMDSBA1_76_63). Notably, a gene cluster present only in AMDSBA3 encodes for a putative squalene biosynthetic pathway (AMDSBA3_32_15-22), including a squalene-cyclase that could be involved in the production of hopanoids [128, 129]. Hopanoids have been previously identified in acidophilic organisms [130], and are thought to alter membrane fluidity and permeability to protons [131].

### Vitamin and coenzyme biosynthesis

Menaquinone biosynthetic pathways were identified in all *Sulfobacillus* genomes, in agreement with detection of a menaquinone with 7 isoprenoid units in *Sulfobacillus thermotolerans* [11]. Complete biosynthetic pathways for lipoic acid, coenzyme A, nicotinamide adenine dinucleotide, flavin adenine dinucleotide, and biotin were identified. Vitamin B12 biosynthetic proteins were found in all organisms except AMDSBA3 and AMDSBA4. All *Sulfobacillus* genomes have B12-dependent methylmaloynl-CoA genes, and thus all likely require the B12 cofactor. The corrinoid-transporting enzyme encoded by the *btuF* gene was found in all species, thus AMDSB3 and AMDSBA4 probably scavenge vitamin B12 from the environment while the other *Sulfobacillus* can either scavenge or synthesize B12 *de novo*.

### Environmental stress

Genes encoding alkyl hydroperoxide reductases/peroxiredoxins and superoxide dismutases were found in all species. Oxidative stress responses are important in all aerobic Bacteria and Archaea, and defense against reactive oxygen species in metal-rich acidic environments is especially important due to redox-active metals creating reactive-oxygen species via the Fenton reaction [132]. Thioredoxin reductases were also identified and may play an important role in reactive oxygen defense as identified in acidophilic *Leptospirillum* bacteria [133].

Genes encoding for the biosynthetic pathways of ectoine and hydroxyectoine were found solely in AMDSBA1 (AMDSBA1_21_55-58). In all *Sulfobacillus*, trehalose could be synthesized by the combined actions of trehalose-6-phosphate synthase and trehalose-6-phosphate phosphatase. However, a second trehalose biosynthetic pathway, involving trehalose synthase [134], was found in *S. thermosulfidooxidans* as well as AMDSBA1, AMDSBA2, and AMDSBA5. Compatible solutes play important roles in adaptation to the osmotic stress induced by the high-ionic strength environments like those found in metal-rich, acidic environments.

All *Sulfobacillus* isolates are known spore formers [10, 11, 13], and all genomes analyzed here are consistent with sporulation capability.

### Transport

Transporters for citrate, potassium, phosphate were identified in all species as were chromate, copper, and arsenite efflux pumps. Numerous amino acid permease-like proteins, oligopeptide and dipeptide transporters, and sugar ABC transport proteins were identified in all species, consistent with a heterotrophic mode of carbon assimilation. Polar amino acid ABC transporters were found only in AMDSBA1, AMDSBA4, and both *S. acidophilus* species and branched amino-acid transport systems were found in all but AMDSBA3. A putative taurine transporter was identified in all but AMDSAB3 and AMDSBA4. Taurine has been identified as a high-abundance compatible solute putatively synthesized by Eukaryotes in the Richmond Mine AMD system, and may provide an important source of nitrogen, carbon, and sulfur for *Sulfobacillus* organisms [135].

### Motility

All *Sulfobacillus* contained operons for the synthesis of flagella and chemotaxis signal transduction systems. Notably, both *S. acidophilus* strains contain nine separate methyl-accepting chemotaxis proteins involved in the transduction of environmental signals to motility response [136]. The other genomes contained far fewer, with AMDSBA2 containing six; AMDSBA3 containing four; and *S. thermosulfidooxidans*, AMDSBA5, AMDSBA4 and AMDSBA1 all containing two or less.

## Discussion

**Figure 5:**
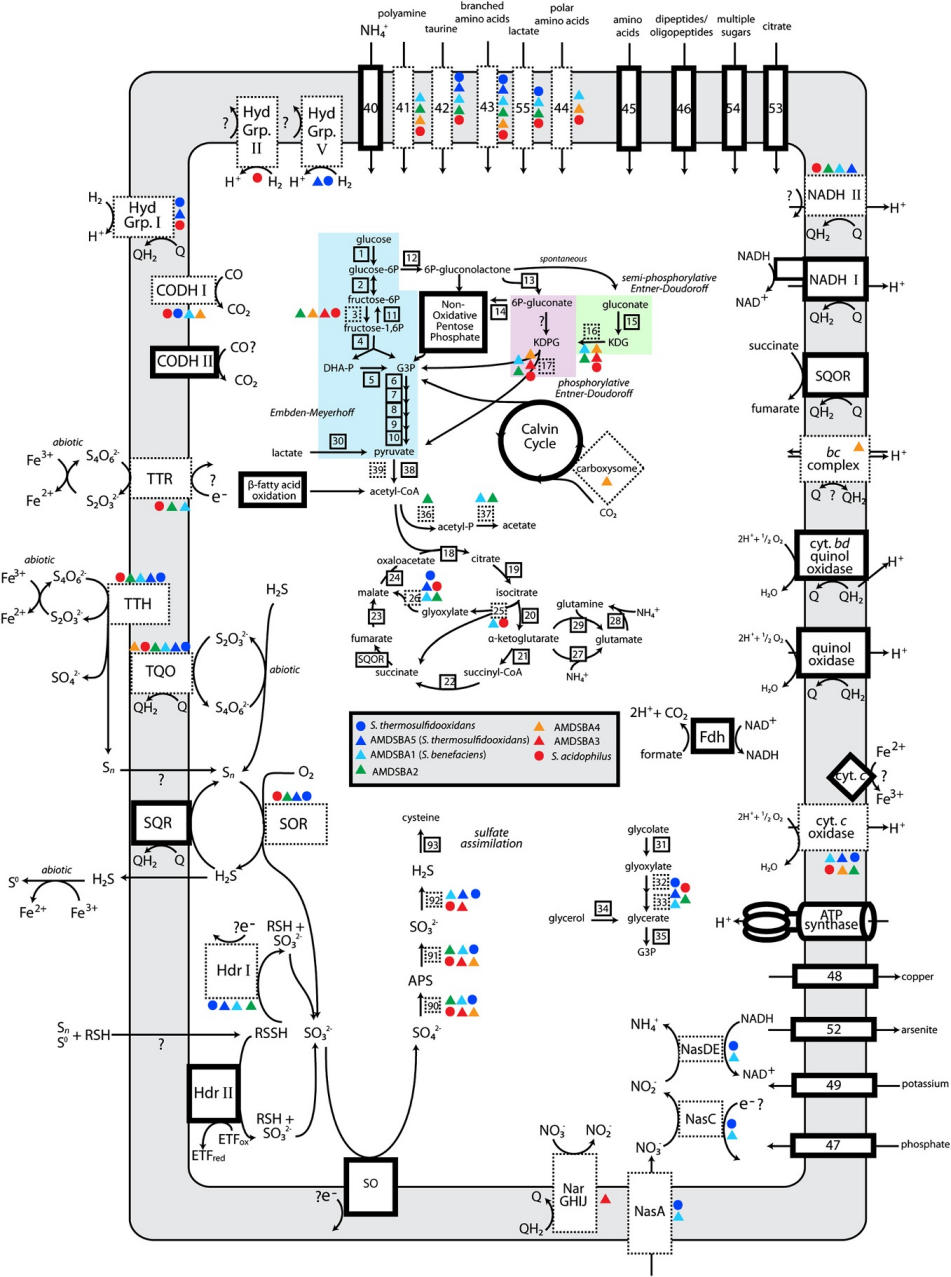
**Reconstruction of central metabolism of** ***Sulfobacillus*** **species.** Dark bordered boxes represent enzymes or enzyme complexes found in all species, while boxes bordered by dashed lines represent those present in only a subset. Numbers correspond to genes listed in Additional file 5: Table S2. Colored circles (previously published genomes) and triangles (AMDSBA genomes) represent which organisms those enzymes are found in, as indicated in the figure legend. CODH, carbon monoxide dehydrogenase complexes; DHA-P, dihydroxyacetone phosphate; Fdh, formate dehydrogenase; G3P, glyceraldehyde-3-phosphate; Hdr, heterodisulfide reductase complexes; KDG, 2-keto-3-deoxygluconate; KDPG, 2-keto-3-deoxygluconate 6-phosphate; SQR, sulfide:quinone oxidoreductae; SQOR, succinate:quinone oxidoreductase; SO, sulfite oxidase; TTH, tetrathionate hydrolase; TQO, thiosulfate:quinone oxidoreductase; TTR, tetrathionate reductase.

Analysis of the *Sulfobacillus* genomes illuminates metabolic pathways used in various transformations of carbon, nitrogen, and sulfur metabolism (summarized Figure 5 and Additional file 5: Table S2). Moreover, potential new metabolisms not yet observed in isolate cultures are potentially encoded within these genomes, including hydrogen oxidation and nitrate reduction.

Carbon fixation via the Calvin cycle is a well-described characteristic of *Sulfobacillus* species [114], and type I RuBisCO genes were identified in all *Sulfobacillus* genomes. It has been noted that *Sulfobacillus* growth rates increase when they are cultivated in the presence of yeast extract and simple sugars [102, 137]. Optimum growth under mixotrophic conditions using inorganic electron donors (usually ferrous iron) and organic carbon (usually glucose or yeast extract) is widely observed [102, 138–140]. To our knowledge, only one isolated strain (*S. acidophilus* NAL) is capable of purely heterotrophic growth, although growth rates are greatly decreased [1]. In another strain of *S. acidophilus* (ALV)*,* it was estimated that about 20% of cellular carbon was derived from CO_2_ fixation during growth on glucose [139].

Prior work demonstrated that glucose consumption proceeded via the oxidative pentose phosphate pathway [139], and that enzymes of the Entner-Doudoroff pathway (6-phosphogluconate dehydratase and 2-keto-3-deoxy-6-phosphogluconate aldolase) were not present. While our results are consistent with the absence of 6-phosphogluconate dehydratase in all genomes studied here, the 2-keto-3-deoxy-6-phosphogluconate aldolase was found in several AMDSBA genomes as well as in the genomes of *S. acidophilus* strains TPY and NAL—allowing for a complete semi-phosphorylative Entner-Doudoroff pathway. The presence of this enzyme in *S. acidophilus* NAL may be a key component contributing to its ability to grow heterotrophically.Across all genomes analyzed here, only two of the enzymes involved in sulfur oxidation were conserved: sulfide:quinone oxidoreductases (SQR) and the heterodisulfide reductase cluster II (Hdr II). While the Hdr II proteins were only present in a single copy amongst all genomes, the SQR proteins were found in between three and six copies for all organisms except AMDSBA1, which contained only one copy (Figure 3). Although the precise reason for the multiple copies of the SQR is unclear, it suggests the importance of sulfide oxidation for many of these organisms.

Sulfide, along with sulfite, can be formed aerobically through a disproportionation reaction of elemental sulfur with sulfur oxygenase (SOR). The subsequent oxidation of sulfide by sulfide:quinone reductase (SQR) allows for the disproportionation reaction of SOR to be coupled to the electron transport chain. While all organisms contain SQR proteins, AMDSBA1, AMDSBA3 and AMDSBA4 lack the SOR proteins necessary to disproportionate sulfur. For these organisms, the source of hydrogen sulfide is unclear, although it may be derived from anaerobic sulfur disproportionation. In this pathway, electrons from sulfane oxidation at the heterodisulfide reductase complex could be used to reduce sulfur, as was proposed for *A. ferroxidans* [99].

Hydrogen utilization, to our knowledge, has also never been experimentally observed in any *Sulfobacillus* species. The presence of several different uptake hydrogenase complexes, however, suggests that hydrogen may be an important source of low-potential electrons for these organisms. In the AMDSBA genomes, only AMDSBA5 contained hydrogenases and these complexes may be a key component of niche specialization for AMDSBA5 in the Richmond Mine. The source of hydrogen, however, is unclear, although flammable and odorless bubbles of gas have been observed to be trapped within the biofilms found in the mine (unpublished observations).

Thiosulfate has also been detected in sediments as well as in solutions within the Richmond Mine [141]. Thiosulfate is predicted to be the first aqueous reaction product of pyrite oxidation [8, 60]. However, because thiosulfate is readily oxidized by ferric iron in acidic media, it is predicted to be found only on the surface of pyritic sediments in the presence of ferric iron. Indeed, thiosulfate was found in bulk solution only in highly reduced conditions essentially devoid of Fe^3+^ [141]. The availability of thiosulfate may explain why *Sulfobacillus* spp. are found so infrequently in the floating biofilms of the Richmond Mine, and are sometimes associated with the sediment or as members of submerged biofilms [7]. Tetrathionate, formed by the oxidation of thiosulfate by ferric iron, is far more stable at acidic pH [142], and thus it is surprising that it was not detected in AMD solutions in the Richmond Mine [8]. The absence of tetrathionate, however, can be explained if it is rapidly oxidized by *Sulfobacillus* organisms.

Amongst all of the genomes analyzed here, AMDSBA4 is unique in many respects. In addition to the putative carboxysome proteins, AMDSBA4 possesses the only *bc* complex amongst all of the *Sulfobacillus*. This is significant as the *bc* complex has been implicated in reverse electron transport during aerobic iron oxidation in *Acidothiobacillus* and *Leptospirillum* species [94]. Furthermore, a second succinate dehydrogenase and a unique cytochrome *c*-oxidase suggest that there may be many distinct aspects of electron transport in AMDSBA4 that warrant further investigation.

## Conclusions

The genomic comparisons presented here advance our understanding of the metabolic potential within the genus *Sulfobacillus* and demonstrate diverse ecological strategies for these organisms. Pathways involved in inorganic sulfur, hydrogen, and nitrogen metabolisms are distributed unevenly amongst species, suggesting key differences in energy metabolism that may be foundational to niche differentiation. Common to all *Sulfobacillus* genomes, however, is a high degree of metabolic versatility, enabling these species to survive in a wide range of acidic environments and adapt to changing biogeochemical conditions. Information gained from this study will help unravel the ecological function of these organisms and their interactions with other organisms in both natural and industrial environments.

### Availability of supporting data

Raw reads were deposited in the NCBI Sequence Read Archive under accession number SRA:SRR191843. Genomes are available at http://ggkbase.berkeley.edu/.

## Electronic supplementary material

Additional file 1: Figure S1: Genome completeness as estimated by the number of conserved single copy genes identified in each of the *Sulfobacillus* genomes. (TIFF 5 MB)

Additional file 2: Table S1: 16S rRNA gene similarity (across 1,124 aligned positions) for published and reconstructed *Sulfobacillus* species. Note that AMDSBA 16S rRNA gene sequences are EMIRGE-generated. (XLSX 22 KB)

Additional file 3: Figure S2: FISH images of AB Muck submerged biofilm showing *Sulfobacillus* (SUL230 probe, red) and archaeal (ARC15 probe, green) populations. (TIFF 5 MB)

Additional file 4: Figure S3: *Sulfobacillus* abundance estimated as a percentage of basepairs mapped to each organism over total sequenced DNA in each sample. 5way fungal streamer data is removed in (B) to better visualize low-abundance organisms. Sample and growth stage is depicted on the X-axis, with GS0-1 indicating low growth-stage biofilms, and GS1.5-2 indicating more mature, thicker growth stage biofilms. (TIFF 8 MB)

Additional file 5: Table S2: Gene loci for central metabolisms discussed in text for all *Sulfobacillus* species. Column “number on figure” refers to numbers indicated in metabolic reconstruction in Figure 5. (XLSX 73 KB)

Additional file 6: Figure S4: Phylogenetic analysis of sulfide:quinone oxidoreductases proteins (SQR). Sequences from *Sulfobacillus* genomes are listed in red. Diamonds indicate nodes with >90% bootstrap support. Bootstrap values greater than 55% are shown as text. Asterisks indicate proteins containing all three conserved active site cysteine residues, all other *Sulfobacillus* sequences contain only the second and third residues. Protein tree adapted from SQR phylogeny laid out by Marcia et al. [77]. The tree is rooted midway to the sulfur oxygenase reductase from *Thioalkalivibrio nitratireducens* (YP 007217840), which was used as an outgroup. (TIFF 17 MB)

Additional file 7: Figure S5: Phylogenetic analysis of tetrathionate hydrolase proteins (TTH). Sequences from *Sulfobacillus* genomes are listed in red. Diamonds indicate nodes with >90% bootstrap support. Bootstrap values greater than 55% are shown as text. Protein tree adapted from Protze et al. [86]. (TIFF 12 MB)

Additional file 8: Figure S6: Phylogenetic analysis of DMSO-reductase superfamily of proteins. Sequences from *Sulfobacillus* genomes are listed in red. Diamonds indicate nodes with >90% bootstrap support. Bootstrap values greater than 55% are shown as text. NapA/NasA, Periplasmic nitrate reductaes; Fdh, formate dehydrogenase; FdnG, formate-hydrogen lyase; NarG, dissimilatory nitrate reductases; EdbR/DdhA/SerA ethylbenzene dehydrogenase/dimethyl sulfide dehydrogenase/selenate reductase; DmsA, dimethyl-sulfoxide reductase; PsrA/PhsA, polysulfide/thiosulfate reductase; SreA, sulfur reductase, SoeA, sulfite-oxidase; TtrA, tetrathionate reductase. (TIFF 19 MB)

Additional file 9: Figure S7: Phylogenetic analysis of hydrogenase superfamily of proteins. Sequences from *Sulfobacillus* genomes are listed in red. Diamonds indicate nodes with >90% bootstrap support. Bootstrap values greater than 55% are shown as text. (TIFF 17 MB)

Additional file 10: Figure S8: Phylogenetic analysis of RuBisCO proteins. Sequences from *Sulfobacillus* genomes are listed in red. Diamonds indicate nodes with >90% bootstrap support. Bootstrap values greater than 55% are shown as text. (TIFF 18 MB)
