# Supplementary figures and images for: Comparison of environmental and isolate Sulfobacillus genomes reveals diverse carbon, sulfur, nitrogen, and hydrogen metabolisms
Source: BMC Genomics. 2014 Dec 15;15:1107. doi: 10.1186/1471-2164-15-1107 (PMC4378227; doi:10.1186/1471-2164-15-1107)

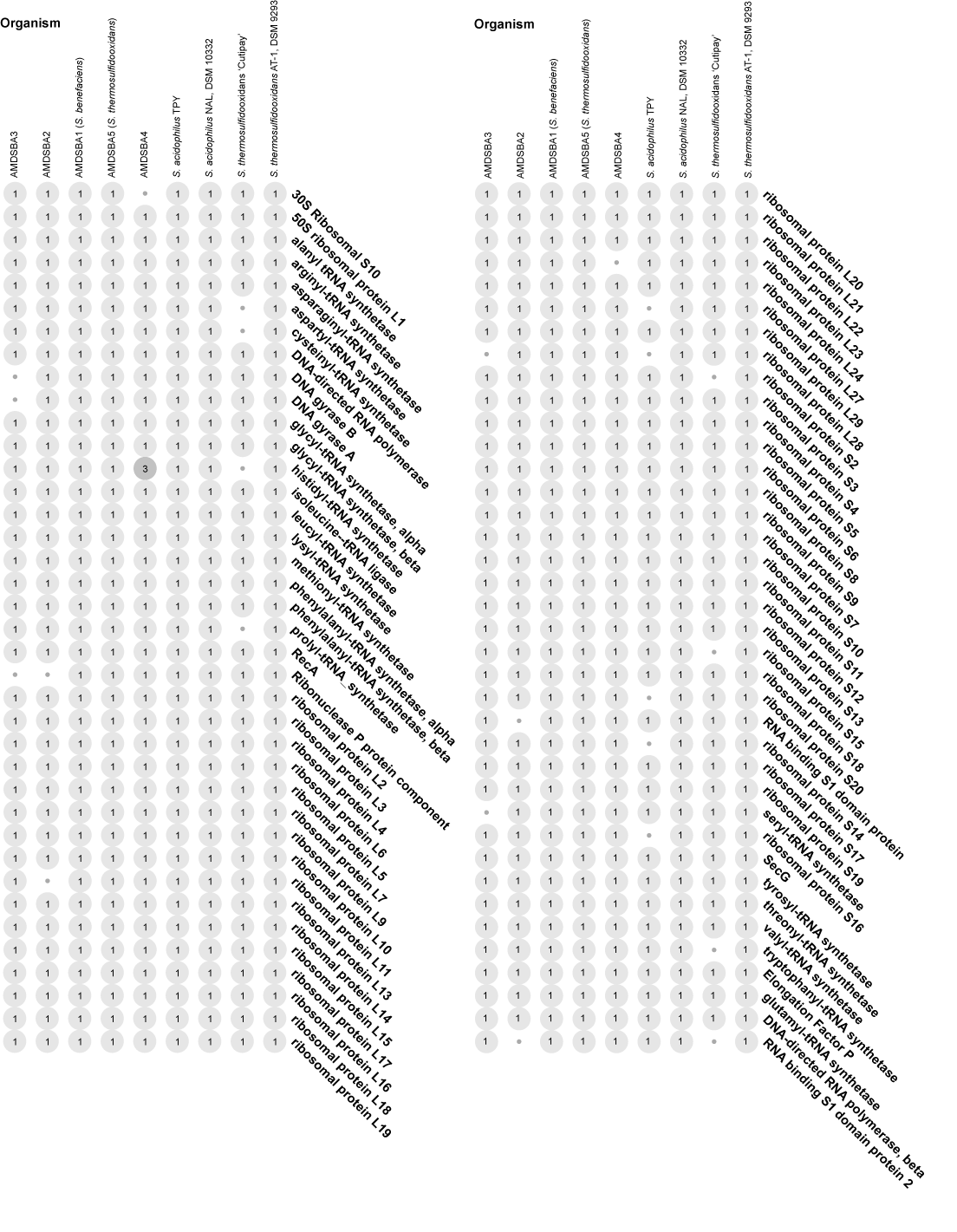

Supplement: Supplementary file 1 — Additional file 1: Figure S1: Genome completeness as estimated by the number of conserved single copy genes identified in each of the Sulfobacillus genomes. (TIFF 5 MB) [file 12864_2014_6919_MOESM1_ESM.tiff]

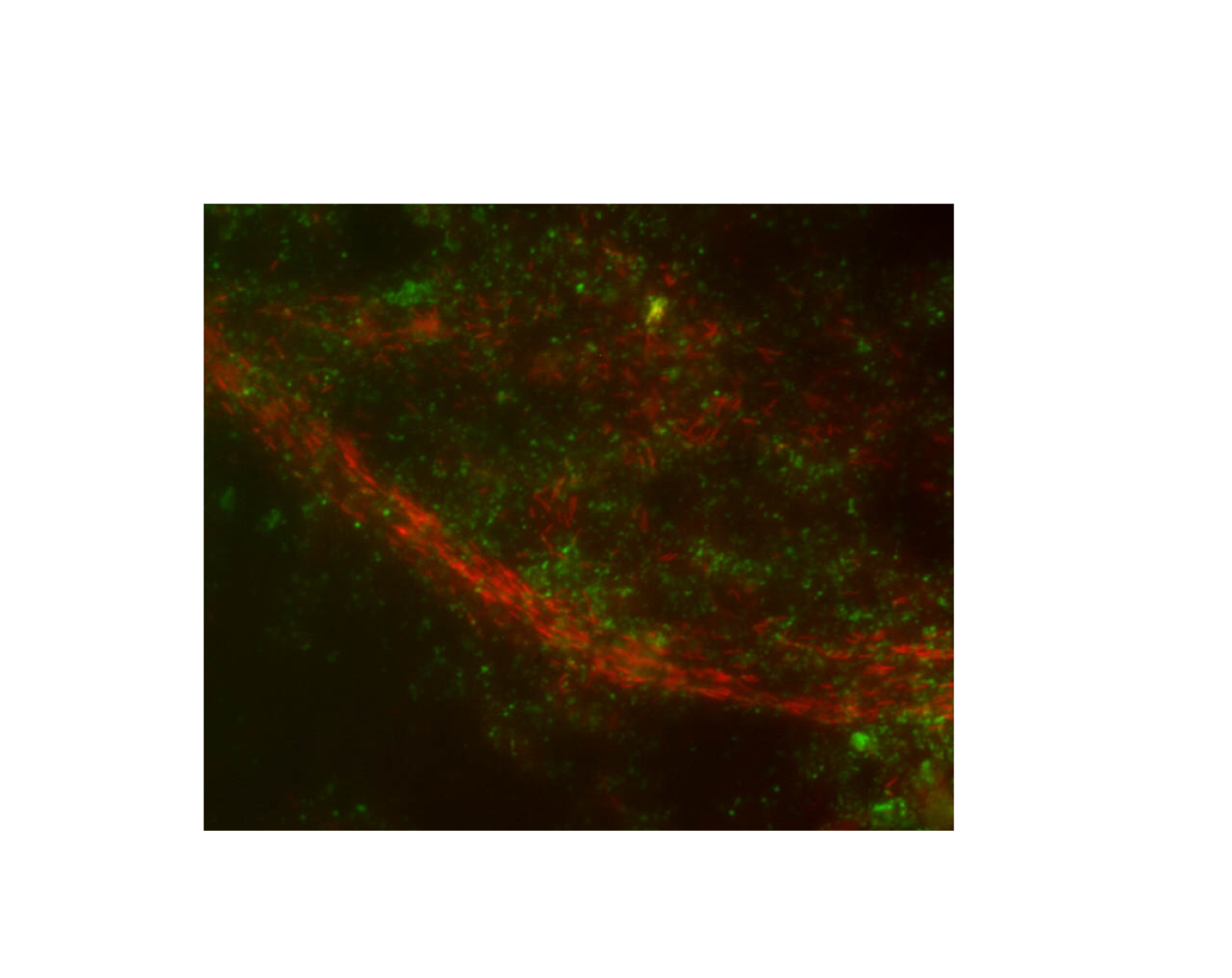

Supplement: Supplementary file 3 — Additional file 3: Figure S2: FISH images of AB Muck submerged biofilm showing Sulfobacillus (SUL230 probe, red) and archaeal (ARC15 probe, green) populations. (TIFF 5 MB) [file 12864_2014_6919_MOESM3_ESM.tiff]

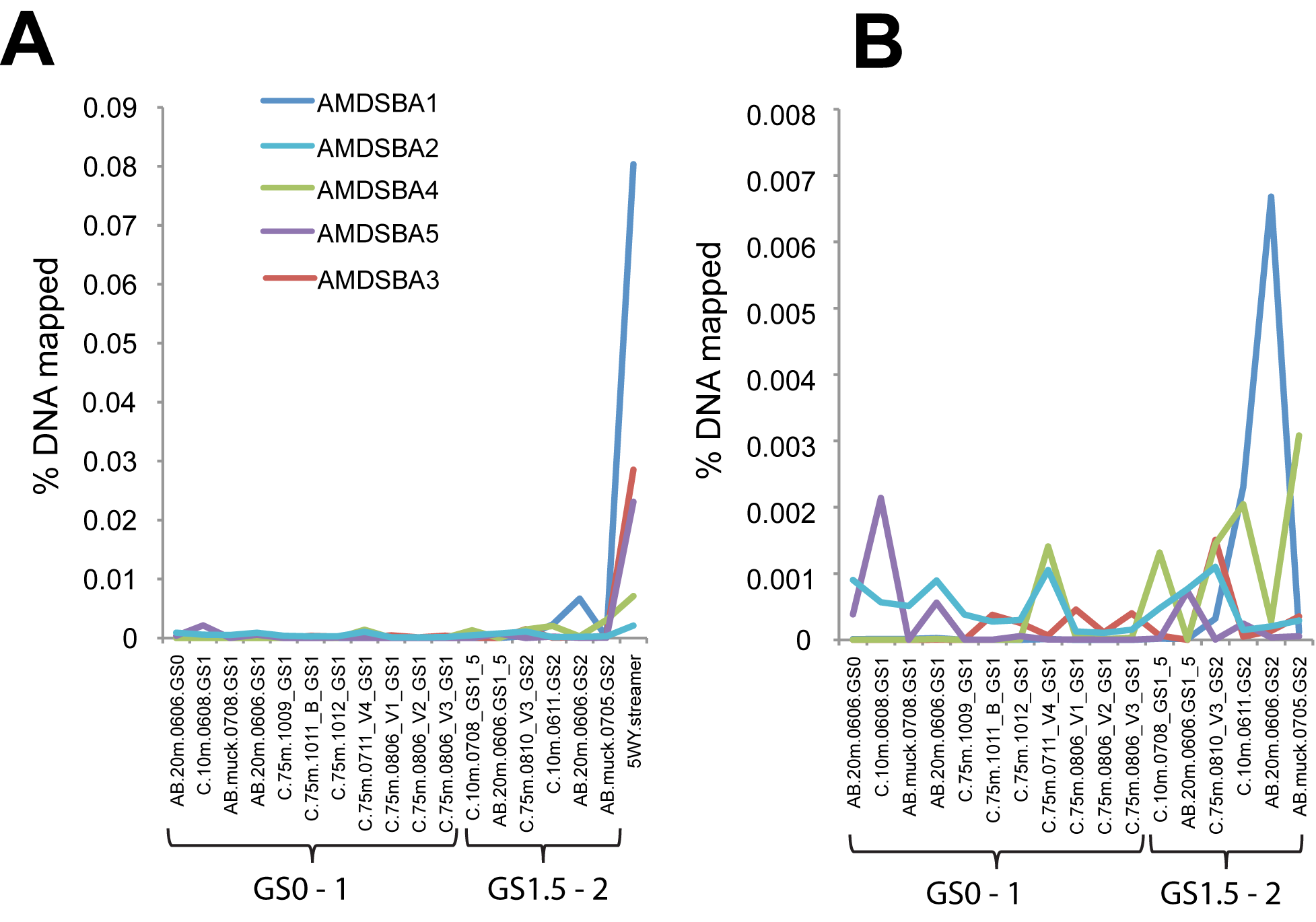

Supplement: Supplementary file 4 — Additional file 4: Figure S3: Sulfobacillus abundance estimated as a percentage of basepairs mapped to each organism over total sequenced DNA in each sample. 5way fungal streamer data is removed in (B) to better visualize low-abundance organisms. Sample and growth stage is depicted on the X-axis, with GS0-1 indicating low growth-stage biofilms, and GS1.5-2 indicating more mature, thicker growth stage biofilms. (TIFF 8 MB) [file 12864_2014_6919_MOESM4_ESM.tiff]

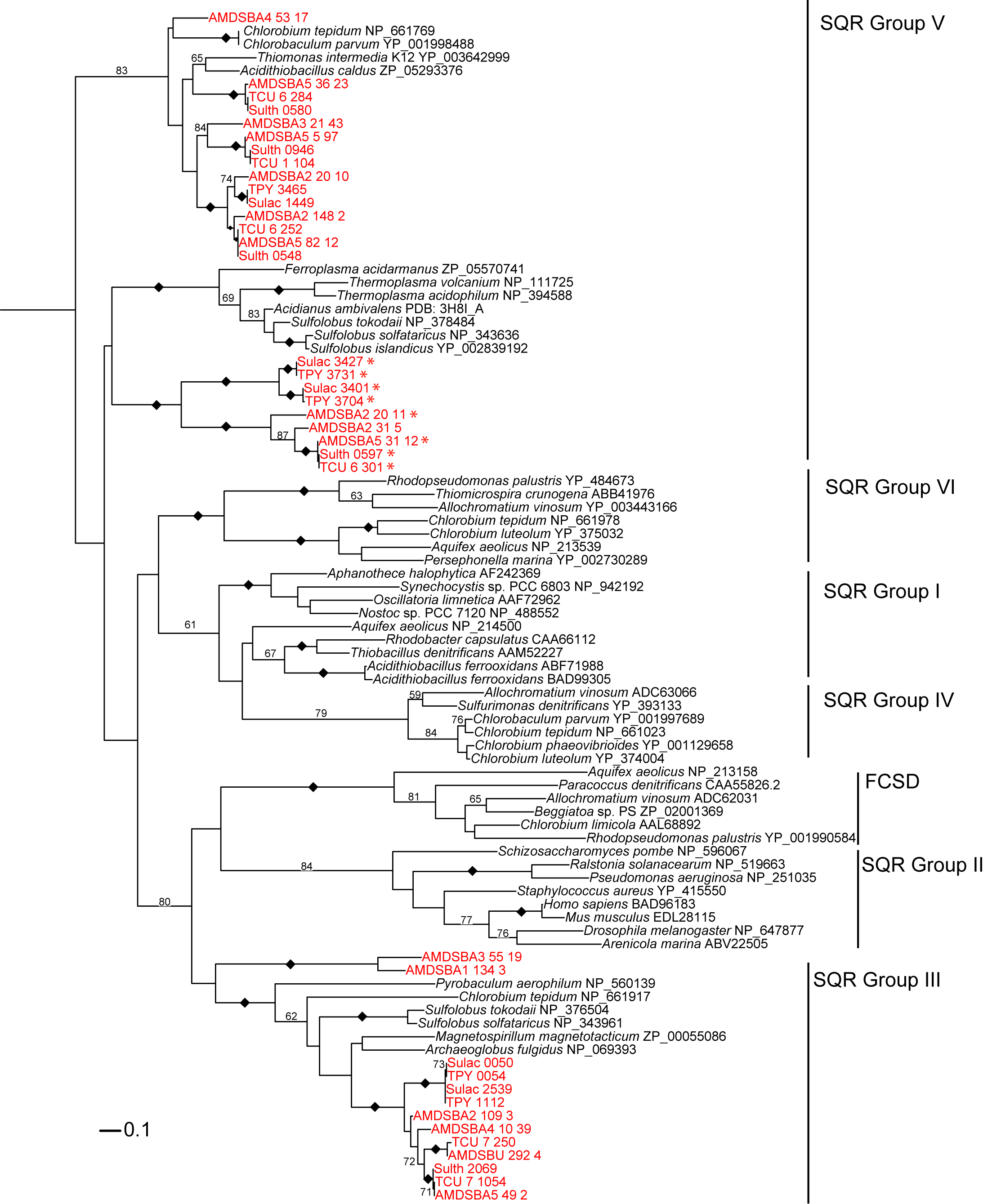

Supplement: Supplementary file 6 — Additional file 6: Figure S4: Phylogenetic analysis of sulfide:quinone oxidoreductases proteins (SQR). Sequences from Sulfobacillus genomes are listed in red. Diamonds indicate nodes with >90% bootstrap support. Bootstrap values greater than 55% are shown as text. Asterisks indicate proteins containing all three conserved active site cysteine residues, all other Sulfobacillus sequences contain only the second and third residues. Protein tree adapted from SQR phylogeny laid out by Marcia et al. [77]. The tree is rooted midway to the sulfur oxygenase reductase from Thioalkalivibrio nitratireducens (YP 007217840), which was used as an outgroup. (TIFF 17 MB) [file 12864_2014_6919_MOESM6_ESM.tiff]

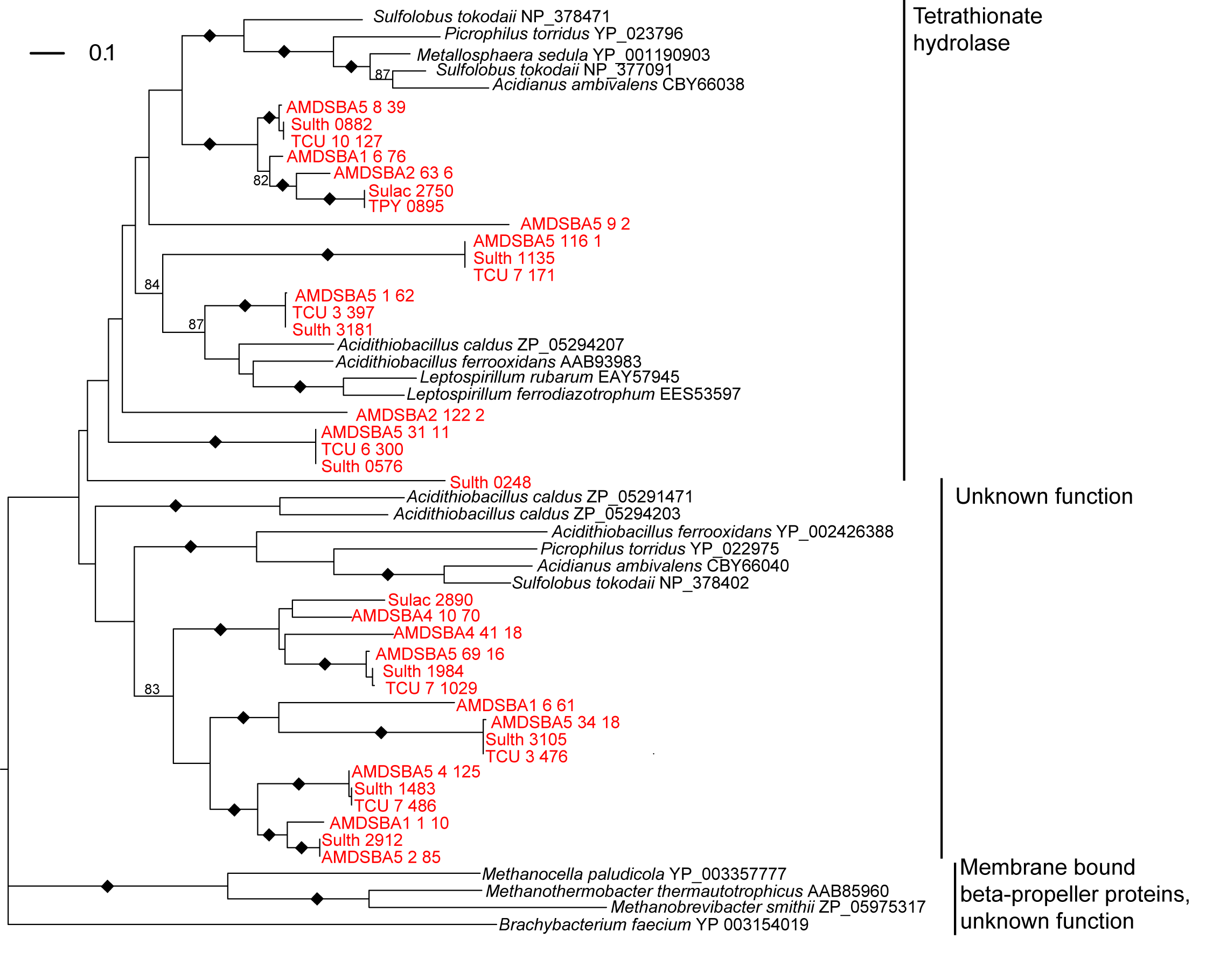

Supplement: Supplementary file 7 — Additional file 7: Figure S5: Phylogenetic analysis of tetrathionate hydrolase proteins (TTH). Sequences from Sulfobacillus genomes are listed in red. Diamonds indicate nodes with >90% bootstrap support. Bootstrap values greater than 55% are shown as text. Protein tree adapted from Protze et al. [86]. (TIFF 12 MB) [file 12864_2014_6919_MOESM7_ESM.tiff]

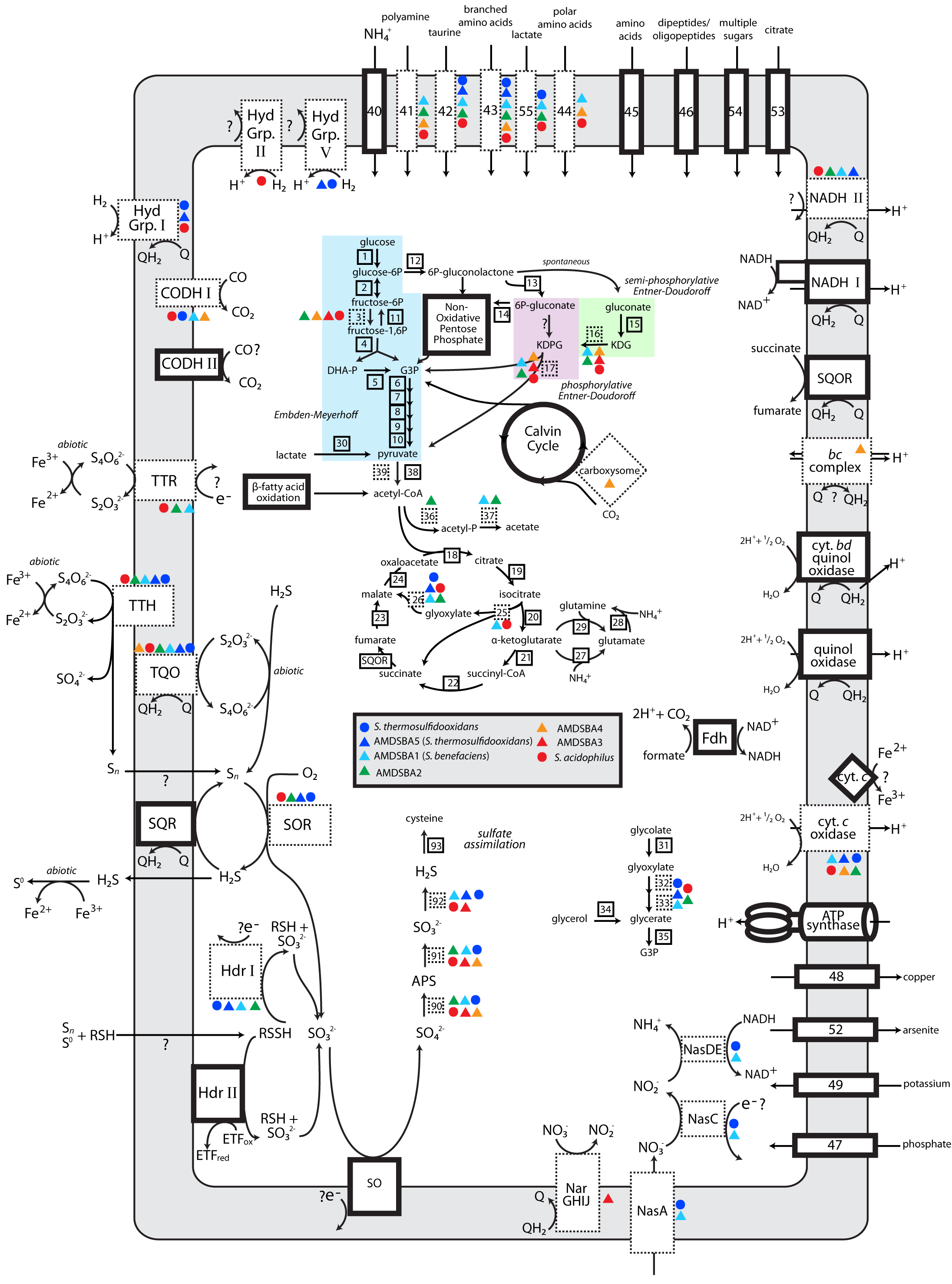

Supplement: Supplementary file 8 — Additional file 8: Figure S6: Phylogenetic analysis of DMSO-reductase superfamily of proteins. Sequences from Sulfobacillus genomes are listed in red. Diamonds indicate nodes with >90% bootstrap support. Bootstrap values greater than 55% are shown as text. NapA/NasA, Periplasmic nitrate reductaes; Fdh, formate dehydrogenase; FdnG, formate-hydrogen lyase; NarG, dissimilatory nitrate reductases; EdbR/DdhA/SerA ethylbenzene dehydrogenase/dimethyl sulfide dehydrogenase/selenate reductase; DmsA, dimethyl-sulfoxide reductase; PsrA/PhsA, polysulfide/thiosulfate reductase; SreA, sulfur reductase, SoeA, sulfite-oxidase; TtrA, tetrathionate reductase. (TIFF 19 MB) [file 12864_2014_6919_MOESM8_ESM.tiff]

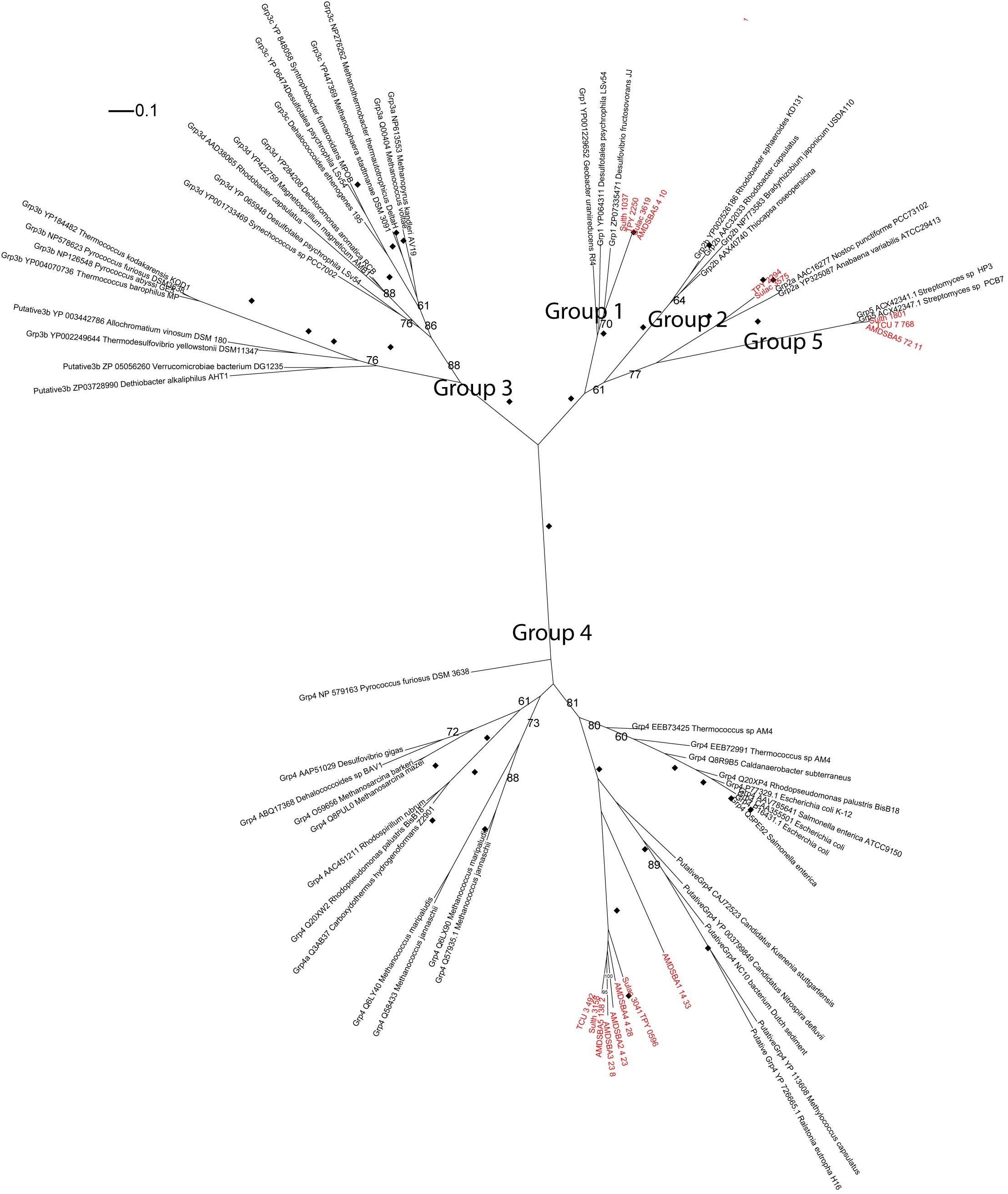

Supplement: Supplementary file 9 — Additional file 9: Figure S7: Phylogenetic analysis of hydrogenase superfamily of proteins. Sequences from Sulfobacillus genomes are listed in red. Diamonds indicate nodes with >90% bootstrap support. Bootstrap values greater than 55% are shown as text. (TIFF 17 MB) [file 12864_2014_6919_MOESM9_ESM.tiff]

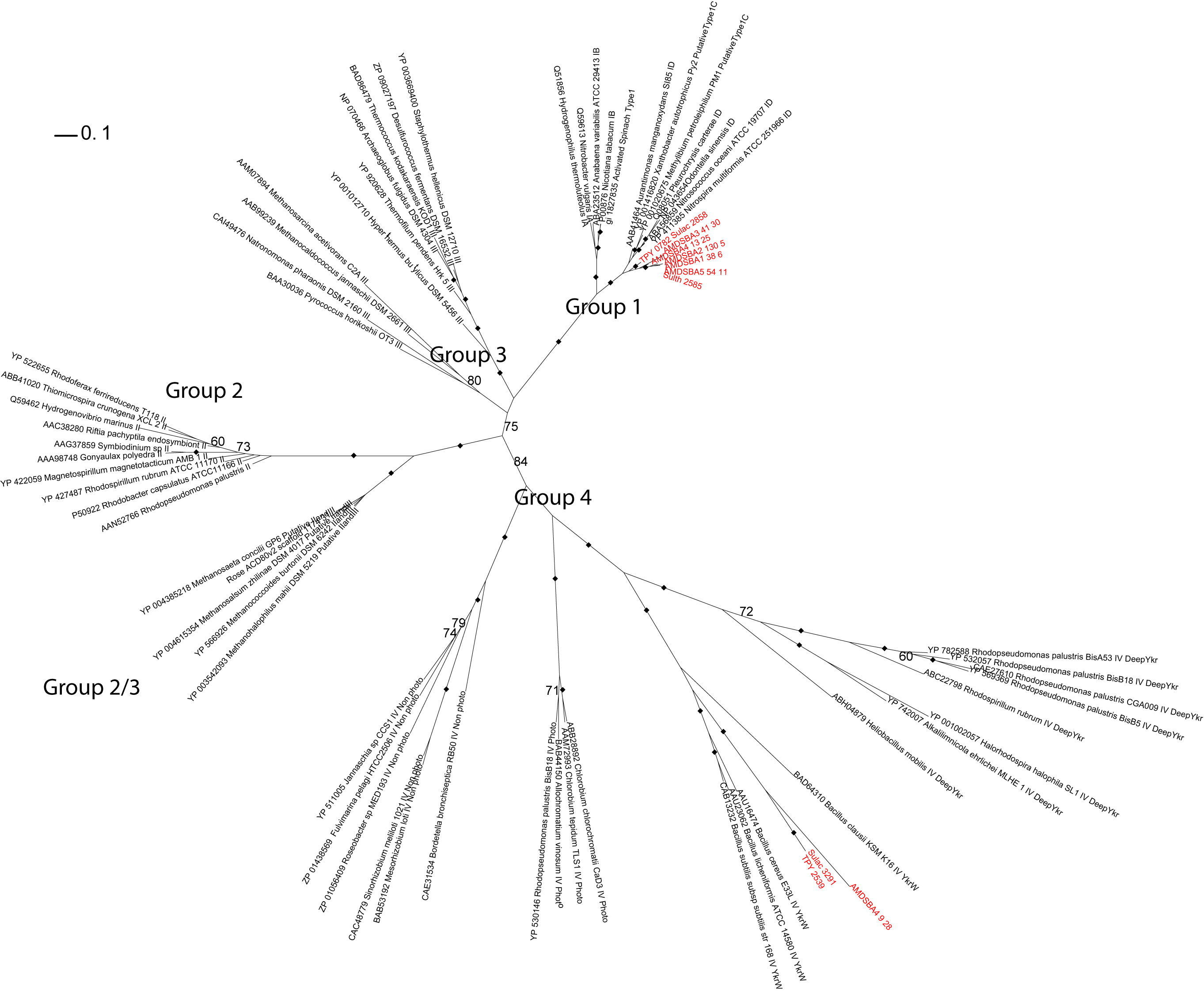

Supplement: Supplementary file 10 — Additional file 10: Figure S8: Phylogenetic analysis of RuBisCO proteins. Sequences from Sulfobacillus genomes are listed in red. Diamonds indicate nodes with >90% bootstrap support. Bootstrap values greater than 55% are shown as text. (TIFF 18 MB) [file 12864_2014_6919_MOESM10_ESM.tiff]
